# Association of Dietary Insulin Index and Load With Glucose Homeostasis Markers, C‐Peptide and Novel Metabolic Indices (TyG‐BMI and METS‐IR) in Physically Active Young Population

**DOI:** 10.1002/edm2.70382

**Published:** 2026-11-03

**Authors:** Negin Nikrad, Nazila Farrin, Mahdieh Abbasalizad Farhangi

**Affiliations:** ^1^ Department of Community Nutrition, Faculty of Nutrition Tabriz University of Medical Sciences Tabriz Iran; ^2^ Nutrition Research Center Tabriz University of Medical Sciences Tabriz Iran; ^3^ Molecular Medicine Research Center Tabriz University of Medical Sciences Tabriz Iran

**Keywords:** C‐peptide, dietary insulin index (DII), dietary insulin load (DIL), glycemic profile, METS‐IR, physically active young population, TyG‐BMI

## Abstract

**Background:**

DII and DIL reflect the insulinemic potential of diet, but their associations with glucose homeostasis, C‐peptide and metabolic indices in physically active young adults are unclear.

**Methods:**

This cross‐sectional study included 221 apparently healthy, physically active adults aged 18–35 years recruited from sports clubs in Tabriz, Iran. Dietary intake was assessed using a validated food frequency questionnaire, and DII and DIL were calculated. Anthropometric, demographic and biochemical parameters were measured. Multiple linear regression models were used to evaluate associations of DII and DIL tertiles with fasting blood sugar, insulin, HOMA‐IR, QUICKI, C‐peptide, METS‐IR and TyG‐BMI, adjusting for potential confounders.

**Results:**

In fully adjusted models, participants in the highest DIL tertile had significantly lower METS‐IR compared with the lowest tertile (β = −1.12; 95% CI: −2.19, −0.05; *p* = 0.04). In multivariable analysis, lower C‐peptide levels were observed in the second DIL tertile (β = −0.63; 95% CI: −1.25, −0.19; *p* = 0.04), whereas the third tertile (*p* = 0.15) and the overall trend across tertiles (*p*‐trend = 0.09) were not statistically significant. DII showed no significant associations with most metabolic markers, except for a borderline trend with QUICKI.

**Conclusion:**

DIL may be more closely related than DII to glycemic profile in physically active young adults. These findings suggest that regular physical activity may attenuate adverse metabolic responses to diets with higher insulinemic load.

## Graphical abstract

In the present cross‐sectional study among 221 physically active young adults, the second DIL tertile was associated with lower C‐peptide, while the highest DIL tertile was associated with lower METS‐IR. DII showed no significant associations with most metabolic markers. Overall, DIL may be more closely related to glycemic and metabolic markers than DII.

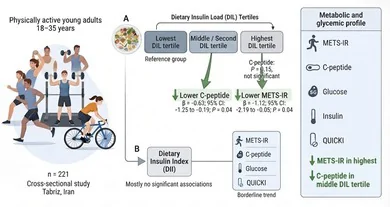

## 1 Introduction

Regular physical activity is firmly established as a cornerstone for improving glucose homeostasis and enhancing the glycemic profile [1]. Efficacy of physical activity primarily linked to its effects on postprandial metabolism that increases the insulin sensitivity of muscles for up to 48 h after exercise [2]. Also, repeated skeletal muscle contractions during physical training stimulate the translocation of glucose transporter type 4 (GLUT4) and increase skeletal muscle glucose uptake and improve peripheral insulin sensitivity [3]. In young, active populations who engage in structured physical activities, these physiological adaptations optimize basal metabolic rates and mitigate tissue‐level insulin resistance [4]. Dietary patterns are key factors affecting glycemic response and insulin sensitivity, which is especially important in physically active populations [5]. Individuals who engage in physical activities often change their eating habits to support their training needs; they usually prefer high‐carbohydrate foods and drinks to improve endurance and performance [6]. However, these dietary requirements lead to significant changes in postprandial metabolism, highlighting the importance of evaluating glycemic and insulinemic responses in this population.

While glycemic index (GI) and glycemic load (GL) are traditional indicators of carbohydrate quality and quantity, they offer a limited capacity for evaluating the total insulinemic impact of a diet [7]. To address this, the dietary insulin index (DII) and dietary insulin load (DIL) were developed to provide a more comprehensive assessment of metabolic responses which focus on the insulin response triggered by all food components, including proteins and fats [7]. The DII quantifies the postprandial insulin response triggered by a wide range of nutrients, including low‐carbohydrate sources such as proteins and specific fats, relative to a reference food [8]. Furthermore, DIL reflects the cumulative insulin demand of the entire diet. This is determined by calculating the sum of the insulin indices for all consumed foods, weighted according to their specific energy contribution [9].

The metabolic impact of a high DII and DIL is primarily characterized by elevated insulin secretion [10]. Evidence from a large cohort study shows that a higher DIL is directly linked to an increased risk of impaired insulin sensitivity over time [11].

To thoroughly evaluate these subtle shifts in insulin sensitivity and beta‐cell function before any overt metabolic dysfunction occurs, it is essential to utilize precise biomarkers, even in healthy and physically active populations. In such demographics, maintaining high insulin sensitivity is key to efficient energy utilization and rapid recovery. The Homeostatic Model Assessment (HOMA‐IR) evaluates baseline glucose homeostasis. Additionally, surrogate markers such as the triglyceride‐glucose body mass index (TyG‐BMI) and the metabolic score for insulin resistance (METS‐IR) provide valuable insights by capturing the dynamic physiological glucose metabolism [12, 13]. Furthermore, accurately assessing endogenous insulin secretion is best achieved through C‐peptide rather than fasting insulin alone. Because C‐peptide does not undergo significant hepatic first‐pass clearance, it offers a highly stable and precise reflection of pancreatic beta‐cell activity [14].

While the metabolic consequences of high DII and DIL are well‐established in sedentary or metabolically compromised groups, their impact on healthy, physically active young adults remains under‐investigated. While regular training improves insulin sensitivity, we hypothesize that diets high in DII and DIL may still impose an excessive metabolic burden. Such dietary stress could trigger subclinical shifts that remain undetected by standard fasting glucose tests due to the masking effect of habitual exercise. Therefore, there is a critical need to clarify how diet‐induced insulin demand interacts with endogenous secretion and resistance markers in athletic populations.

To address this gap, this study examines the relationship between dietary insulin indices (DII and DIL) and key glycemic profile markers, specifically C‐peptide and insulin resistance indices and insulin sensitivity indices in a population of young, physically active adults.

## 2 Methods and Materials

This cross‐sectional study was conducted in Tabriz, Iran, yielding a sample of 221 apparently healthy, physically active young adults aged 18–35 years. A convenience sampling method was employed to recruit participants from various local sports clubs, targeting individuals engaged in exercise modalities such as yoga, Pilates, aerobics and bodybuilding. Sampling procedures and data acquisition for this study took place from June 6, 2022, through August 12, 2023. To be eligible for inclusion, participants were required to be within the specified age range (18–35 years old), express a willingness to participate, and maintain a consistent exercise routine of at least 4 h per week aimed at preserving general health and fitness. Conversely, individuals were excluded if they had a history of substance or alcohol abuse or suffered from chronic illnesses that could interfere with standard dietary intake, such as gastrointestinal disorders or unexplained appetite loss. Furthermore, those with pre‐existing cardiometabolic conditions, including cardiovascular diseases and diabetes, were excluded from the study. This study protocol has been approved by the ethics committee of the Tabriz University of Medical Sciences (Code: IR.TBZMED.REC.1405.304 and grant number: 79662) and was conducted in accordance with the principles of the Declaration of Helsinki. Prior to any data collection, the study objectives were thoroughly explained and written informed consent was obtained from all participants.

### 2.1 Sociodemographic and Physical Activity Assessment

Sociodemographic information was collected through face‐to‐face interviews conducted by trained staff. To evaluate participants' physical activity, the short form of the International Physical Activity Questionnaire (IPAQ) was utilized. This 7‐item questionnaire is a widely recognized tool, and its validity and reliability have been previously confirmed for this population [15].

### 2.2 Anthropometric and Body Composition Measurements

All anthropometric measurements were performed by a trained nutritionist. Body weight was measured to the nearest 0.1 kg using a Seca 753E electronic scale, while participants were wearing light clothing and no shoes. Standing height was measured to the nearest 0.1 cm using a standard stadiometer. Body mass index (BMI) was subsequently calculated using the standard formula: weight in kilograms divided by the square of height in metres (kg/m^2^). Waist circumference (WC) was measured at the narrowest point of the torso, and hip circumference (HC) was measured at the maximum extension of the buttocks; the waist‐to‐hip ratio (WHR) was then calculated by dividing the WC by the HC. Additionally, detailed body composition parameters were evaluated using bioelectrical impedance analysis (BIA) equipment (Tanita, BC‐418 MA, Tokyo, Japan) while participants were lightly dressed.

### 2.3 Dietary Assessment

Habitual dietary intake over the previous year was evaluated using a validated 80‐item Food Frequency Questionnaire (FFQ), which is a condensed version of the standard 168‐item FFQ previously validated for use in the general population [16]. The FFQ was administered by trained nutrition experts through in‐person interviews lasting approximately 45 min per participant. During the interview, the nutritionist read each food item, and participants reported their frequency of consumption on a daily, weekly or monthly basis.

The reported frequencies were multiplied by standard portion sizes or household measurements to determine daily consumption, which was then converted to grams. Portion sizes were standardized based on guidelines confirmed by the United States Department of Agriculture (USDA). Total daily energy and macro‐ and micronutrient intakes were calculated using Nutritionist 4 software.

### 2.4 Calculation of Metabolic Indices

The formulas that were implemented to calculate the indices measured in the present study are indicated in the following [17, 18, 19].

#### 2.4.1 Homeostatic Model Assessment for Insulin Resistance (HOMA‐IR)

HOMA‐IR calculated using fasting glucose and fasting insulin levels:

$$\text{HOMA}-\mathrm{IR}=\frac{\text{Fasting Insulin}\;\left(\mathrm{\mu U}/\mathrm{mL}\right)\times \text{Fasting Glucose}\;\left(\mathrm{mg}/\mathrm{dL}\right)}{405}$$

#### 2.4.2 Quantitative Insulin Sensitivity Check Index (QUICKI)

QUICKI determined by the logarithmic transformation of fasting blood glucose and insulin:

$$\text{QUICKI}=\frac{1}{\log_{10}\left(\text{Fasting Insulin}\;\mathrm{\mu U}/\mathrm{mL}\right)+\log_{10}\left(\text{Fasting Glucose}\;\mathrm{mg}/\mathrm{dL}\right)}$$

#### 2.4.3 Triglyceride‐Glucose Index Multiplied by BMI (TyG‐BMI)

The TyG index, a surrogate marker for peripheral insulin resistance, will first be calculated as follows:

$$\mathrm{TyG}\;\text{Index}=\ln\left(\frac{\text{Fasting Triglycerides}\;\left(\mathrm{mg}/\mathrm{dL}\right)\times \text{Fasting Glucose}\;\left(\mathrm{mg}/\mathrm{dL}\right)}{2}\right)$$

Subsequently, the TyG‐BMI was calculated by multiplying the TyG index by the participant's Body Mass Index (BMI):

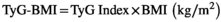




#### 2.4.4 Metabolic Score for Insulin Resistance (METS‐IR)

METS‐IR was calculated combining fasting glucose, triglycerides, HDL cholesterol (HDL‐c) and BMI:

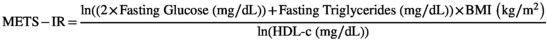




### 2.5 Biochemical Measurements

Venous blood samples (5 mL) were collected from all participants following an 8–12 h overnight fast. Blood was drawn from the antecubital vein by a trained phlebotomist while the participants were seated, strictly adhering to standard health and safety protocols.

The collected blood samples were immediately centrifuged to separate the serum from the whole blood. The extracted serum aliquots were then frozen and stored at −80°C until the time of biochemical analysis.

Serum levels of fasting blood sugar (FBS), triglycerides (TG) and high‐density lipoprotein cholesterol (HDL‐C) were analysed through enzymatic methods using the colorimetric technique by an automated analyser. Serum fasting insulin and C‐peptide levels were quantified using the standard enzyme‐linked immunosorbent assay (ELISA) method, following the general instructions provided by the manufacturers.

### 2.6 Statistical Analysis

The normal distribution of continuous variables was assessed using the Kolmogorov–Smirnov test. Continuous variables with normal distribution were presented as mean ± standard deviation (SD), while categorical variables were expressed as frequencies and percentages. Participants were categorized into three tertiles (T1–T3) based on their dietary insulin index (DII) and dietary insulin load (DIL) scores. To compare the mean differences of baseline characteristics and metabolic indices (FBS, Insulin, HOMA‐IR, QUICKI, C‐Peptide, TyG‐BMI and METS‐IR) across the tertiles of DII and DIL, a One‐Way Analysis of Variance (ANOVA) was utilized.

Multivariable linear regression models were conducted to evaluate the association between the tertiles of DII and DIL (independent variables) and the metabolic/insulin resistance indices (dependent variables). The first tertile (T1) was considered the reference category. Results were reported as unstandardized beta coefficients (β) along with 95% confidence intervals (CI). To control for potential confounders, three models were evaluated: a crude model (Model I), a model adjusted for age, sex, BMI and dietary energy intake (kcal) (Model II), and a model further adjusted for waist‐to‐hip ratio and education level (Model III). Additionally, linear trends across categories (*p*‐trend) were tested by assigning the median value of each tertile to participants and entering this as a continuous variable into the regression models.

## 3 Results

The baseline demographic, anthropometric and lifestyle characteristics of the final 221 study participants, categorized by tertiles of DIL and DII, are presented in Table 1. Participants in the highest tertile of DIL (T3) had a significantly higher proportion of males (*p* < 0.01), a higher ratio of waist‐to‐hip (*p* = 0.04), moreover higher daily energy intake compared to those in the lowest tertile (3250.90 ± 1214.64 and 1795.79 ± 394.45 kcal, respectively and *p* < 0.01). Additionally, the distribution of educational levels differed significantly across DIL tertiles (*p* < 0.01). However, no significant differences were observed in age, weight, BMI, fat mass (FM), fat‐free mass (FFM) or training frequency between tertiles of DIL (*p* > 0.05). Conversely, when participants were categorized according to DII tertiles, no significant differences were found in any of the assessed demographic, anthropometric or lifestyle variables, including age, BMI, fat mass, energy intake and training frequency (all *p* > 0.05).

**TABLE 1:** Baseline demographic, anthropometric and lifestyle characteristics of study participants across tertiles of dietary insulin load (DIL) and dietary insulin index (DII).

Variables	Tertiles of DIL				Tertiles of DII			
	T1 (*N* = 73)	T2 (*N* = 74)	T3 (*N* = 74)	*p*	T1 (*N* = 73)	T2 (*N* = 74)	T3 (*N* = 74)	*p*
Age	23.28 ± 3.58	22.47 ± 3.28	22.98 ± 3.84	0.30*	22.83 ± 3.07	22.77 ± 3.63	23.13 ± 3.99	0.80*
Gender (% male)	38.4	50.00	64.9	**< 0.01** **	56.20	51.40	45.90	0.46**
Weight	64.72 ± 11.98	67.55 ± 13.30	75.46 ± 14.09	0.06*	70.07 ± 14.45	68.38 ± 12.38	69.37 ± 14.80	0.79*
BMI	22.81 ± 3.05	22.82 ± 3.58	24.52 ± 3.56	0.41*	23.26 ± 3.64	23.11 ± 3.01	23.78 ± 3.78	0.48*
WHR	0.78 ± 0.06	0.80 ± 0.06	0.80 ± 0.07	**0.04** *	0.80 ± 0.06	0.78 ± 0.06	0.79 ± 0.07	0.27*
FM	14.39 ± 6.32	14.11 ± 6.33	15.91 ± 7.55	0.72*	14.62 ± 7.36	14.30 ± 6.31	15.49 ± 6.65	0.54*
FFM	50.10 ± 9.52	52.98 ± 10.74	59.13 ± 11.58	0.06*	55.02 ± 11.20	53.80 ± 10.99	53.46 ± 11.66	0.67*
kcal	1795.79 ± 394.45	2288.80 ± 493.46	3250.90 ± 1214.64	**< 0.01** *	2521.23 ± 1010.97	2508.68 ± 1015.39	2315.39 ± 954.39	0.37*
Education (%)								
Under‐diploma and diploma	37.00	39.2	56.80	**< 0.01** **	38.40	48.60	45.90	0.48**
Bachelor	45.2	54.1	29.70		46.60	43.20	39.20	
Post graduate	17.8	6.8	13.50		15.10	8.10	14.90	
Training frequency (days/week)	4.86 ± 1.50	4.12 ± 1.63	4.87 ± 1.62	0.12**	5.08 ± 1.59	4.78 ± 1.58	4.60 ± 1.54	0.18*
MET‐min/week	2169.49 ± 1776.01	2898.00 ± 2237.46	3612.75 ± 3749.36	**< 0.01** *	2995.86 ± 2698.35	2660.81 ± 1867.72	3034.73 ± 3531.05	0.66

*Note:* All data are mean (±SD) except education and gender, that is presented as percent in each group. Bolded values are statistically significant.

Abbreviations: BMI, Body mass index; DII, Dietary Insulin Index; DIL, Dietary Insulin Load; FFM, Fat Free Mass; FM, Fat Mass; WHR, waist‐to‐hip ratio.

* *p* values derived from One‐Way ANOVA with Tukey's post hoc comparisons.

** *p* values derived from chi‐squared test.

The biochemical and metabolic profiles of the study participants across the tertiles of DIL and DII are presented in Table 2. Regarding DIL, in the crude model, participants in the highest tertile of DIL had significantly higher TyG‐BMI compared to those in the lowest tertile (*p* < 0.01), with a significant linear trend across the tertiles (p‐trend < 0.01). However, this association disappeared after adjusting for potential confounders in Models 2 and 3 (*p* = 0.20 and *p* = 0.21, respectively). Other glycemic markers, including fasting blood sugar (FBS), insulin, HOMA‐IR, QUICKI, C‐peptide and METS‐IR, did not show any significant differences across DIL tertiles in either crude or adjusted models (*p* > 0.05).

**TABLE 2:** Metabolic and glycemic characteristics across tertiles of DII and DIL.

Variables	Tertiles of DIL					Tertiles of DII				
	T1 (*N* = 73)	T2 (*N* = 74)	T3 (*N* = 74)	*p*	*p*‐trend	T1 (*N* = 73)	T2 (*N* = 74)	T3 (*N* = 74)	*p*	*p*‐trend
FBS	70.00 ± 7.16	70.37 ± 8.45	70.38 ± 7.27	0.52^a^ 0.92^b^ 0.69^c^	0.26	75.27 ± 8.87	73.37 ± 8.47	74.21 ± 9.64	0.20^a^ 0.25^b^ 0.48^c^	0.88
INSULIN	9.24 ± 4.71	9.42 ± 4.96	8.84 ± 5.65	0.36^a^ 0.38^b^ 0.32^c^	0.19	9.50 ± 7.97	11.24 ± 12.15	8.33 ± 5.48	0.15^a^ 0.10^b^ 0.09^c^	0.64
HOMA‐IR	1.59 ± 0.81	1.67 ± 1.01	1.55 ± 1.02	0.30^a^ 0.35^b^ 0.35^c^	0.15	1.76 ± 1.54	2.06 ± 2.32	1.54 ± 1.05	0.19^a^ 0.12^b^ 0.11^c^	0.64
QUIKI	0.36 ± 0.03	0.36 ± 0.03	0.37 ± 0.03	0.22^a^ 0.21^b^ 0.20^c^	0.18	0.36 ± 0.04	0.36 ± 0.03	0.38 ± 0.07	0.12^a^ 0.05^b^ 0.05^c^	0.26
Peptid‐C	2.32 ± 2.14	1.71 ± 1.64	1.92 ± 1.71	0.13^a^ 0.109^b^ 0.09^c^	0.19	2.05 ± 2.16	2.02 ± 1.83	1.94 ± 1.63	0.73^a^ 0.69^b^ 0.68^c^	0.64
TyG‐BMI	184.11 ± 31.72	191.24 ± 42.12	205.03 ± 38.96	**< 0.01** ^a^ 0.20^b^ 0.21^c^	**< 0.01**	189.44 ± 34.51	188.76 ± 32.37	199.45 ± 41.12	0.35^a^ 0.14^b^ 0.15^c^	0.18
METS‐IR	32.84 ± 6.47	33.54 ± 8.27	35.25 ± 6.06	0.09^a^ 0.09^b^ 0.12^c^	0.08	34.24 ± 6.78	33.46 ± 5.88	35.03 ± 7.13	0.63^a^ 0.64^b^ 0.75^c^	0.67

*Note:* Data are presented as mean ± standard deviation (SD). p‐values for the comparison of continuous variables across tertiles were obtained using Analysis of Variance (ANOVA) for the crude model and Analysis of Covariance (ANCOVA) for adjusted models. p‐trend was calculated by assigning the median value of each tertile as a continuous variable. *p*‐trend, linear trend across tertiles. Bolded values are statistically significant.

Abbreviations: DII, Dietary Insulin Index; DIL, Dietary Insulin Load; FBS, Fasting Blood Sugar; HOMA‐IR, Homeostatic Model Assessment for Insulin Resistance; METS‐IR, Metabolic Score for Insulin Resistance; QUICKI, Quantitative Insulin Sensitivity Check Index; T1–T3, Tertile 1 to Tertile 3; TyG‐BMI, Triglyceride‐Glucose Index related to Body Mass Index.

^a^ Model 1: Crude (unadjusted).

^b^ Model 2: Adjusted for age, sex, Body Mass Index (BMI), Physical activity and total energy intake (kcal).

^c^ Model 3: Further adjusted for Waist‐to‐Hip Ratio (WHR) and education level.

When evaluating DII, no significant differences were observed across the tertiles for most metabolic variables in the crude analysis. After multivariable adjustment in Models 2 and 3, the overall differences in QUICKI across DII tertiles were of borderline statistical significance (*p* = 0.05); however, the trend across tertiles was not statistically significant (*p*‐trend = 0.26). Similarly, insulin and HOMA‐IR levels did not demonstrate any statistically significant linear trends across DII tertiles in the fully adjusted models (Table 2).

Table 3 presents the multivariable linear regression analyses evaluating the associations of DIL and DII tertiles with markers of glucose homeostasis and metabolic indices.

**TABLE 3:** Multivariable linear regression analysis for the association between biochemical variables and tertiles of DIL and DI.

Variable	Model	Tertiles of DIL			p	Tertiles of DII			p
		T1 (*N* = 73)	T2 (*N* = 74) β (CI); p	T3 (*N* = 74) β (CI); p		T1 (*N* = 73)	T2 (*N* = 74) β (CI); p	T3 (*N* = 74) β (CI); p	
FBS (mg/dL)	I	Ref	−1.16 (−4.30, 1.97); 0.46	−1.80 (−4.94, 1.33); 0.26	0.52	Ref	−2.59 (−5.71, 0.53); 0.10	−0.242 (−3.36, 2.88); 0.88	0.20
	II	Ref	−0.65 (−3.85, 2.55); 0.15	−0.47 (−4.39, 3.44); 0.81	0.92	Ref	−2.54 (−5.61, 0.53); 0.10	−0.62 (−3.7, 2.47); 0.69	0.25
	III	Ref	0.38 (−2.42, 3.19); 0.78	1.68 (−1.76, 5.13); 0.33	0.69	Ref	−1.34 (−4.16, 1.48); 0.35	0.31 (−4.16, 1.48); 0.35	0.48
Insulin (mIU/L)	I	Ref	−0.28 (−3.34, 2.78); 0.85	−2.07 (−5.15, 1.00); 0.18	0.36	Ref	2.17 (−0.84, 5.19); 0.15	−0.73 (−3.83, 2.36)	0.15
	II	Ref	−0.35 (−3.46, 2.74); 0.82	−2.56 (−6.45, 1.33); 0.19	0.38	Ref	2.18 (−0.75, 5.11); 0.14	−1.01 (−4.05, 2.02); 0.51	0.10
	III	Ref	−0.46 (−3.56, 2.62); 0.76	−2.84 (−6.73, 1.05); 0.15	0.32	Ref	2.07 (−0.87, 5.02); 0.16	−1.23 (−4.28, 1.80); 0.42	0.09
HOMA‐IR	I	Ref	−0.06 (−0.65, 0.52); 0.82	−0.43 (−1.02, 0.15); 0.15	0.30	Ref	0.37 (−0.20, 0.95); 0.20	−0.14 (−0.73, 0.45); 0.63	0.19
	II	Ref	−0.07 (−0.66, 0.52); 0.81	−0.51 (−1.25, 0.23); 0.17	0.35	Ref	0.38 (−0.18, 0.94); 0.18	−0.20 (−0.79, 0.37); 0.48	0.12
	III	Ref	−0.10 (−0.69, 0.49); 0.73	−0.52 (−1.27, 0.21); 0.16	0.35	Ref	0.39 (−0.17, 0.96); 0.17	−0.21 (−0.80, 0.36); 0.46	0.11
QUICKI	I	Ref	−0.00 (−0.02, 0.01); 0.76	0.01 (−0.00, 0.03); 0.18	0.22	Ref	−0.00 (−0.02, 0.01); 0.35	0.01 (−0.01, 0.03); 0.27	0.12
	II	Ref	−0.00 (−0.02, 0.01); 0.75	0.01 (−0.00, 0.03); 0.19	0.21	Ref	−0.01 (−0.02, 0.01); 0.32	0.01 (−0.00, 0.03); 0.16	0.05
	III	Ref	−0.00 (−0.02, 0.01); 0.79	0.01 (−0.01, 0.04); 0.16	0.20	Ref	−0.01 (−0.02, 0.01); 0.33	0.01 (−0.00, 0.03); 0.13	0.05
C‐peptide	I	Ref	**−0.60 (−1.19, −0.01); 0.04**	−0.39 (−0.98, 0.19); 0.12	0.13	Ref	0.10 (−0.49, 0.69); 0.74	−0.14 (−0.73, 0.45); 0.64	0.73
	II	Ref	**−0.61 (−1.22, −0.00); 0.04**	−0.47 (−1.23, 0.28); 0.21	0.10	Ref	0.15 (−0.45, 0.77); 0.60	−0.09 (−0.72, 0.52); 0.75	0.69
	III	Ref	**−0.63 (−1.25, −0.19); 0.04**	−0.47 (−1.23, 0.28); 0.21	0.09	Ref	0.15 (−0.45, 0.76); 0.61	−0.10 (−0.72, 0.52); 0.74	0.68
TyG‐BMI	I	Ref	2.69 (−8.54, 13.94); 0.63	**18.89 (7.64, 30.13); < 0.01**	**< 0.01**	Ref	1.25 (−10.25, 12.76); 0.83	7.93 (−3.57, 19.44); 0.17	0.35
	II	Ref	3.17 (−0.67, 7.01); 0.10	3.75 (−0.94, 8.46); 0.11	0.20	Ref	3.12 (−0.58, 6.84); 0.09	3.46 (−0.27, 7.21); 0.07	0.14
	III	Ref	3.09 (−0.77, 6.95); 0.11	3.72 (−0.98, 8.43); 0.12	0.021	Ref	3.10 (−0.63, 6.85); 0.10	3.40 (−0.35, 7.15); 0.07	0.15
METS‐IR	I	Ref	−0.28 (−2.33, 1.76); 0.78	1.83 (−0.21, 3.87); 0.07	0.09	Ref	−0.55 (−2.62, 1.50); 0.59	0.44 (−1.61, 2.51); 0.67	0.63
	II	Ref	−0.28 (−1.16, 0.60); 0.53	**−1.18 (−2.26, −0.10); 0.03**	0.09	Ref	−0.25 (−1.12, 0.60); 0.55	−0.41 (−1.28, 0.44); 0.34	0.64
	III	Ref	−0.29 (−1.18, 0.58); 0.50	**−1.12 (−2.19, −0.05); 0.04**	0.12	Ref	−0.14 (−1.01, 0.71); 0.73	−0.33 (−1.19, 0.53); 0.44	0.75

*Note:* Data are expressed as unstandardized regression coefficients (β) and 95% Confidence Intervals (CIs) using multivariable linear regression models. The lowest tertile (T1) served as the reference category. *p* trend, linear trend across tertiles. Model I: Crude (unadjusted). Model II: Adjusted for age, sex, Body Mass Index (BMI), Physical activity and total energy intake (kcal). Model III: Further adjusted for Waist‐to‐Hip Ratio (WHR) and education level. Bolded values are statistically significant.

Abbreviations: DII, Dietary Insulin Index; DIL, Dietary Insulin Load; FBS, Fasting Blood Sugar; HOMA‐IR, Homeostatic Model Assessment for Insulin Resistance; METS‐IR, Metabolic Score for Insulin Resistance; QUICKI, Quantitative Insulin Sensitivity Check Index; Ref, Reference; TyG‐BMI, Triglyceride‐Glucose Index related to Body Mass Index.

Regarding DIL, in the crude model (Model I), participants in the highest tertile had a significantly higher TyG‐BMI compared to those in the lowest tertile (β=18.89; 95% CI: 7.64,30.13; p<0.01), with a significant positive linear trend across tertiles (*p*‐trend < 0.01). However, this association was attenuated and lost statistical significance after adjusting for potential confounders in Models II and III. Conversely, individuals in the second tertile of DIL exhibited significantly lower C‐peptide levels compared to the reference group across all models (Model III: β = −0.63; 95% CI: −1.25, −0.19; *p* = 0.04), although the overall linear trend was not statistically significant (*p* > 0.05). For METS‐IR, a significant inverse association was observed in the highest tertile of DIL in Model II (β = −1.18; 95% CI: −2.26, −0.10; *p* = 0.03), which remain significant in the fully adjusted model (β = −1.12; 95% CI: −2.19, −0.05; *p* = 0.04). Other markers, including FBS, insulin and HOMA‐IR, did not show any significant associations with DIL tertiles (*p* > 0.05). When evaluating DII, no significant associations were observed for FBS, insulin, HOMA‐IR, C‐peptide, TyG‐BMI and METS‐IR across the tertiles in both crude and adjusted models. For QUICKI, while the highest tertile did not show a statistically significant difference compared to the reference group, a significant borderline linear trend was observed across DII tertiles in the adjusted models (Models II and III *p*‐trend = 0.05).

## 4 Discussion

In this cross‐sectional study of active young adults, we investigated the associations of DIL and DII with markers of glucose homeostasis and novel metabolic indices. The inverse association between DIL and C‐peptide was limited to the second tertile, and the absence of a significant trend suggests that this finding should be interpreted as non‐linear. Furthermore, higher DIL was independently and inversely associated with the METS‐IR. Notably, while a crude positive association was observed between DIL and TyG‐BMI, this relationship became non‐significant after adjustment, underscoring the strong confounding effects of baseline characteristics. However, DII was not significantly associated with most metabolic parameters. The borderline trend for QUICKI after multivariable adjustment should be interpreted cautiously because the individual tertile estimates were not statistically significant.

Our finding that DIL is a more robust predictor of metabolic fluctuations than DII is highly consistent with recent epidemiological evidence, particularly in active populations. In line with our results, Beba et al. [20] demonstrated that DIL was significantly associated with multiple body composition parameters (percent body fat and body mass index) in professional soccer players, whereas DII showed negligible associations. This stronger predictive utility of DIL has also been observed in general populations; for instance, Elyasi et al. [9] reported a significant association between DIL and the risk of Metabolic Syndrome (MetS) in women, while DII failed to show a similar independent effect. Furthermore, Khoshnoudi‐Rad et al. [21] found that high adherence to both DIL and DII was strongly linked to long‐term weight gain and increased MetS risk in weight‐stable women. The stronger association of DIL across these studies can be attributed to its methodological advantage: while DII solely reflects carbohydrate quality, DIL accounts for both the quality and the total absolute quantity of the dietary intake, thereby providing a more accurate estimation of the true postprandial insulin demand. Moreover, our observation regarding the modulation of C‐peptide levels by DIL holds significant clinical relevance. As established by Sokooti et al. [14], elevated baseline plasma C‐peptide is a powerful, independent predictor of incident type 2 diabetes. Therefore, the capacity of DIL to capture early alterations in markers like C‐peptide and METS‐IR emphasizes its utility over DII in assessing real‐world metabolic risks.

In this study, novel indices such as METS‐IR and TyG‐BMI were utilized because these indices integrate fasting gluco‐lipid profiles with anthropometric measures to provide a highly sensitive assessment of early metabolic deterioration [22]. The clinical importance of evaluating these indices in young, physically active populations is underscored by recent evidence; Lin et al. [23] demonstrated that TyG derivatives are independently associated with subclinical cardiac decline and reduced exercise capacity even in young, active military personnel, highlighting their unique utility in detecting hidden metabolic risks. Furthermore, the profound sensitivity of these indices to lifestyle and dietary modifications has been widely established. Font et al. [24] and Du et al. [25] confirmed that dietary quality and physical activity levels are critical, interacting determinants of METS‐IR and TyG‐BMI scores. Similarly, Moulavi et al. [26] reported significant correlations between diet‐related parameters and TyG‐BMI, suggesting that diet composition strongly modulates these combined lipid‐adiposity indices. DIL dictates postprandial insulin surges which subsequently drive both lipogenesis and adiposity; METS‐IR and TyG‐BMI serve as exact translational surrogate markers to capture these specific pathways. Results of the present study demonstrated a significant inverse association between DIL and METS‐IR, with an association with TyG‐BMI. This inverse relationship suggests that in physically active youths, a higher dietary insulin demand may be modulated or metabolized differently after carefully adjusting for overall energy intake and central adiposity (WHR), that a complex metabolic interaction that these multifaceted indices successfully brought to light.

Several plausible physiological mechanisms may explain the findings of the present study. Under normal physiological conditions, diets characterized by a high DIL provoke excessive insulin secretion, eventually culminating in compensatory hyperinsulinemia [8]. This hyperinsulinemic state upregulates lipogenic pathways and deteriorates insulin sensitivity, which is often reflected by a decline in QUICKI. However, a distinct metabolic pattern is observed in physically active youth, where skeletal muscle serves as the predominant primary site for glucose utilization [27]. In this population, repeated muscle contractions induce insulin‐independent glucose uptake, a process primarily mediated by the AMP‐activated protein kinase (AMPK) signalling pathway and the subsequent translocation of GLUT4 transporters to the cell membrane [3]. Consequently, these profound physiological adaptations within the muscular tissue can significantly alleviate the secretory burden on pancreatic beta‐cells [28]. Such mechanisms can effectively attenuate, or even reverse, the anticipated adverse impacts of a high‐DIL diet, providing a robust biological rationale for the non‐significant associations of the DII and the inverse relationships observed in our fully adjusted models (Figure 1).

**FIGURE 1:**
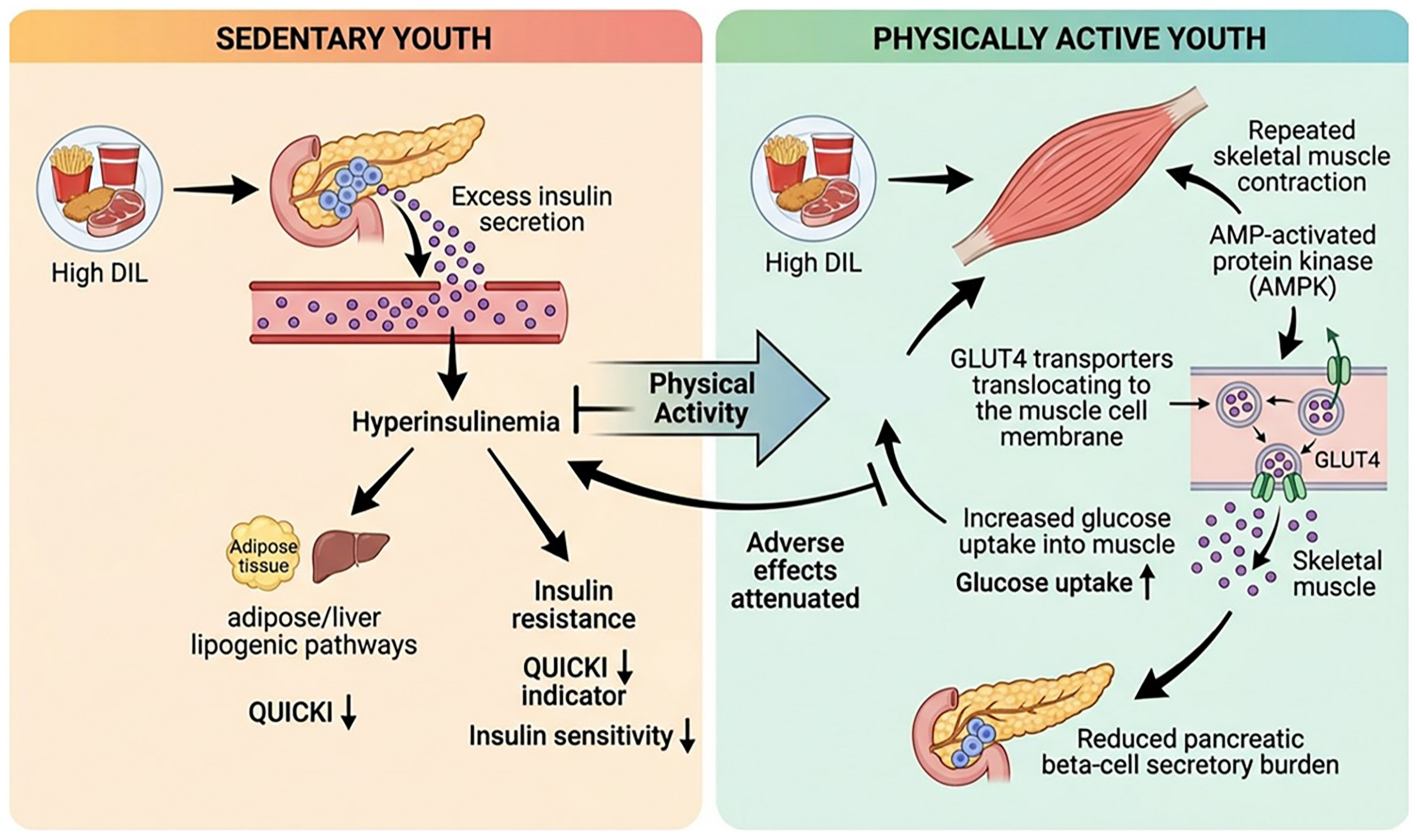
High DIL affects glycemic metabolism in sedentary versus physically active youth. (This graphical abstract was generated with the assistance of the Noah AI image‐generation tool. The authors supplied the prompt, reviewed the output and took full responsibility for the final figure content.) Because the present study exclusively enrolled physically active young adults without an internal sedentary control group, the depicted pathway for sedentary youth represents a theoretical mechanistic concept synthesized from existing literature rather than an empirical observation of this study.

The present study has several strengths. First, to our knowledge, this is the first study evaluating the insulinemic potential of the diet in a specific and understudied population of physically active youth. This focus provides novel insights into how regular physical activity might modulate diet‐induced metabolic responses. Second, we utilized novel, cost‐effective and highly accurate indices of insulin resistance, such as TyG‐BMI, METS‐IR and QUICKI, which offer superior prognostic value compared to traditional isolated markers. Third, the simultaneous assessment of both the DII and DIL allowed for a comprehensive evaluation of both the quality and the overall quantity of the diet's insulinemic potential. Fourth, the assessment of serum C‐peptide levels alongside fasting insulin is a major methodological advantage. C‐peptide serves as a more reliable and stable marker of endogenous insulin secretion, given its negligible hepatic extraction and longer half‐life compared to insulin. Finally, the rigorous statistical approach, which included multiple multivariable linear regression models, enabled robust adjustments for a wide array of potential confounders.

Despite these strengths, several limitations must be acknowledged. Primarily, the cross‐sectional design of the study prevents any causal inferences regarding the relationships between DIL/DII and metabolic indices. Future well‐designed prospective cohort studies are warranted to confirm these temporal associations. Furthermore, dietary intake was assessed using a Food Frequency Questionnaire (FFQ); while validated, self‐reported dietary data are fundamentally susceptible to recall bias and measurement error, which may lead to the misclassification of dietary exposures. Lastly, although we adjusted for numerous covariates in our final models, the possibility of residual confounding from unmeasured or inadequately measured genetic, environmental, or lifestyle factors cannot be entirely ruled out.

## 5 Conclusion

In conclusion, a higher DIL was significantly and inversely associated with METS‐IR in active young adults, whereas the association with C‐peptide was non‐linear and not consistent across tertiles. Conversely, the DII did not exhibit robust associations with the assessed metabolic indices. Given the unique physiological and metabolic adaptations inherent to physically active young adults, these associations are specific to this demographic and should not be generalized to sedentary individuals or older populations. Further prospective cohort studies focused on active populations are required to elucidate the long‐term clinical relevance of these dietary patterns on metabolic health.

## Author Contributions

**Negin Nikrad:** methodology, writing – review and editing, writing – original draft, conceptualization, investigation. **Nazila Farrin:** writing – original draft, writing – review and editing, formal analysis, data curation. **Mahdieh Abbasalizad Farhangi:** conceptualization, methodology, writing – original draft, investigation, supervision.

## Funding

We thank all of the study participants. This study protocol has been approved by the ethics committee of the Tabriz University of Medical Sciences (Code IR.TBZMED.REC.1405.304 and grant number: 79662).

## Consent

The authors have nothing to report.

## Conflicts of Interest

The authors declare no conflicts of interest.

## Data Availability

The data that support the findings of this study are available on request from the corresponding author. The data are not publicly available due to privacy or ethical restrictions.
